# Body image and self-perception in women with navel piercings

**DOI:** 10.1371/journal.pone.0274099

**Published:** 2022-09-09

**Authors:** Christine Coleman, Helge Gillmeister

**Affiliations:** Department of Psychology and Centre for Brain Science, University of Essex, Colchester, United Kingdom; University of Turin, ITALY

## Abstract

The present study investigated how women’s body image and body-perceptual processes are affected by navel piercings, an embellishment of the abdominal region women often feel negatively about. We probed perceptual (response times), cognitive (surveys), affective (aesthetic ratings) and neural (event-related potentials, ERPs) facets of (own) body perception. We found that navel piercings are primarily motivated by the desire to enhance one’s body image, and can significantly improve bodily self-perception relative to before and to imagined removal of the piercing. Hence, body image concerns in women with navel piercings were found to be comparable to those of a control group; and their aesthetic ratings of other women’s abdomens only differed, positively, for images depicting navel piercings. ERPs indicated that the sight of navel piercings enhances early structural encoding of bodies as well as late emotional-motivational processes, especially in women with navel piercings. We further found a strong self-advantage in both cortical and behavioural responses during recognition of own and others’ abdomens, especially for images displaying the piercing. Altogether, findings suggest that navel piercings become strongly, and beneficially, integrated into women’s bodily self image. Such piercings may thus be seen as expressions of body care that can protect against self-harming thoughts and behaviours.

## 1. Introduction

Body piercing dates back at least 5300 years and has ancestral roots across many different cultures. It is believed that body piercing marked significant spiritual or developmental stages [1] or indicated status and wealth [2]. For example, navel piercings were a rite of passage for Ancient Egyptian pharaohs [2]. However, additional meanings ascribed to body piercings have arisen over time. Van Hoover et al., [1] highlighted piercing “for self-expression, for aesthetic value, for sexual pleasure, and to conform to societal norms or to rebel against them” (p.521).

The rebellious stigma associated with body piercing stemmed from the social movements in the 1970s and 1980s, in which body piercings were used as markers of group identity to rebel against conservative middle-class norms [1, 3]. However, from the 1990s onwards, body piercing became more conventional because of its embellishing features and the increasing representation of body modifications in the media [1, 4]. Anywhere between 6.5% and 51% of the population have body piercings, with the specific percentage depending on which age group and social grouping is targeted [e.g., 5; for review see 6, 7]. Moreover, Van Hoover et al., [1] noted that 50% of millennials have at least one piercing in locations other than the earlobes, thus indicating the ever-growing popularity of this type of body modification.

Much of the scientific literature on body piercings has focused on the associated negative implications, such as medical complications, risk-taking behaviour and adverse personality traits [e.g. 8, 9]. Individuals with piercings are still frequently subjected to various forms of stigmatisation [1, 10], which disproportionately affects those whose self-image is more strongly determined by their piercings [10]. The most common stigma is the association with rebellious behaviours, including drug use and other risk-taking behaviours, criminal tendencies and sociological instability [11–14, see also 6]. However, given the growing popularity and normalisation of body piercings, studies have begun to turn their attention to the more extensive and complex motivations underlying body piercing [e.g., 3, 7, 15]. This is important because an understanding of how something as simple as a piercing can influence people’s attitudes and evaluations of themselves and others, can provide a basis for eliminating negative perceptions within society.

Common reasons for getting a body piercing are the desire to ornament one’s own body, increasing sexual attractiveness, enhancing one’s individuality, being a member of a subculture, friendship or love, fashion, adapting to one’s environment, and commemorating an important moment [e.g., 6, 7, 13]. This indicates that body piercings can be strongly linked to body image and one’s sense of self as an individual.

The existing literature on body piercings has included several or all piercing sites [1]. To the best of our knowledge, no study has investigated the motivations or effects of one particular site in depth. After the ears, navels are consistently the most common site for women to have pierced [e.g., 1, 7, 16, 17]. At the same time, investigations into women’s body image have highlighted the abdominal region as a body part that women are most sensitive about and unhappy with [e.g., 18–20, see also 21] and thus either overly attend to [22] or avoid [21, 23, but see 24]. For these reasons, the present study selectively investigates navel piercings in women. We hypothesised that navel piercings positively affect body image and self perception [see also 7, 25].

We used a multi-method approach, applying cognitive (surveys), affective (aesthetic ratings), perceptual (speeded response task) and neuroscientific (event-related potentials) tools to delineate in some depth how women with and without navel piercings experience their own and others’ pierced and unpierced bodies. The reason for this approach lies in the inherent complexity of the body image construct. Our body image, the conscious mental representation of our own body [26], is not a singular dimension of experience but consists of several facets: cognitive (what we think about our body), affective (what we feel about our body), perceptual (how we perceive our body), and behavioural (what kind of behaviours we engage in if we are dissatisfied with our body) [e.g., 18, 27, see also 28]. All of these facets are thought to be affected in body image disturbances, such as those contributing to the symptomatology of eating and body dysmorphic disorders [see 28–30], which also remain more prevalent in women than in men [e.g., 31, 32].

Body image-related reasons (desire for body ornamentation or embellishment, increased physical or sexual attractiveness) are listed among the reported motivations for piercings in general [1, 6, 7], but they do not typically dominate over other frequently cited motivations [individual self-expression, rebelliousness; 1, 5, 6, 13, 33]. One exception is the most recent large-scale survey of body piercings [7], which found that embellishment of the body was the most common motivation among women and one that distinguished women from men. We therefore hypothesised that body image-related reasons would heavily outweigh other reasons why women obtain navel piercings. We also expected women to report that their navel piercings improved their body image and self perception. Specifically we expected individuals’ sense of identity (body ownership and agency aspects of self-awareness, ie., feeling “themselves” and in control of body and actions), perceived success and attractiveness, satisfaction with body shape and size as well as with the sight of their stomach, and comfort with looking at themselves and with other people looking at them, to have increased following their navel piercing relative to before the piercing, and to decrease again with imagined temporary removal of their piercing. We also expected women to agree with two additional statements: One probed hatred of their abdominal region, because women with navel piercings are attempting to embellish an area of the body they often feel negatively about [e.g., 19]. The second probed feelings of fortuitous events occurring as a result of their navel piercing, because individuals would feel more attractive and, subsequently, more self-confident [34] after their navel piercing. Since we hypothesised that navel piercings improve body image, we also measured body dysmorphic concerns [35]. We predicted that women with navel piercings would, as a result of their piercing, have no more concerns than women without navel piercings.

In addition to cognitive (survey) tools, we measured aesthetic perception and self-recognition of bodies in brain and behaviour. We expected that aesthetic ratings of (other) women’s bodies (abdominal region only) would be higher when the images included a navel piercing compared to identical images not including a navel piercing, especially in participants with navel piercings, who would perceive them as embellishing [1, 7]. We further expected women to be faster and more accurate at recognising their own body than a stranger’s body (abdominal region only), in line with the self-advantage found for faces [for a recent review see 36] and other body parts [37–39]. Critically, if navel piercings are part of individuals’ identity and self-image, we expected this self-advantage to be larger for images of bodies with than without navel piercings.

Finally, we also expected the corresponding visual event-related potentials (ERPs) to be affected by the presence of navel piercings during aesthetic perception and self-recognition tasks in a subset of participants with and without navel piercings. The ERP components of interest in this study were early cortical P1-N1 components [associated with attention and discriminative processing including body-structural encoding and self-recognition; e.g., 40–46], mid-latency component N250 [associated with self- and general identity recognition; e.g., 47, 48], and the late positive potential [LPP; associated with emotional-evaluative and motivational processes including aesthetics; e.g., 49, 50]. We expected enhancement of the P1-N1 complex (indicating self-advantage in body-structural encoding), and the N250 (indicating identity recognition) for images of the self vs. other women in the self-recognition task. In both tasks P1-N1 and LPP were also expected to be enhanced for bodies with vs. without piercings, because piercings might engage early attentional processes reflected at P1-N1 [e.g., 44, 46] as well as late emotional-motivational processes reflected at LPP [e.g., 49, 51]. This was expected to be the case especially for pierced participants, for whom piercings are more salient, emotionally relevant, and an integral part of the body.

## 2. Method

### 2.1. Participants

A total of 344 participants with and without navel piercings, were recruited using a variety of methods. Locally, we used email advertisements, posters and a participant recruitment database at the University of Essex. Social networking was used to recruit pierced and non-pierced participants more widely and consisted of recruitment via Survey Circle and Survey Tandem, Facebook posts by the first author that were shared on by friends and family, as well as the endorsement of our study by an art historian who specialises in body modification as artistic practice and is well-connected to international body modification communities. Participants recruited for the EEG task were local to the University of Essex and received £15 in remuneration, while all others volunteered or received course credits. All participants were female, with the majority of participants residing in the United Kingdom (66.9%), 8.1% in South Africa, 7.3% in other countries, and 17.7% who did not indicate their country of residence.

There were 214 female participants with navel piercings, with a mean age of 25.7 years (SD: 6.7, range: 18–55). Of the 202 participants who indicated how often they wear their navel piercing, 87.6% indicated that they always wear their navel piercing, 4.5% stated most of the time, 1.5% about half of the time and 6.4% indicated sometimes. The control sample consisted of 130 female participants without navel piercings, with a mean age of 25.6 years (SD: 7.0, range: 18–48).

**Fig 1:**
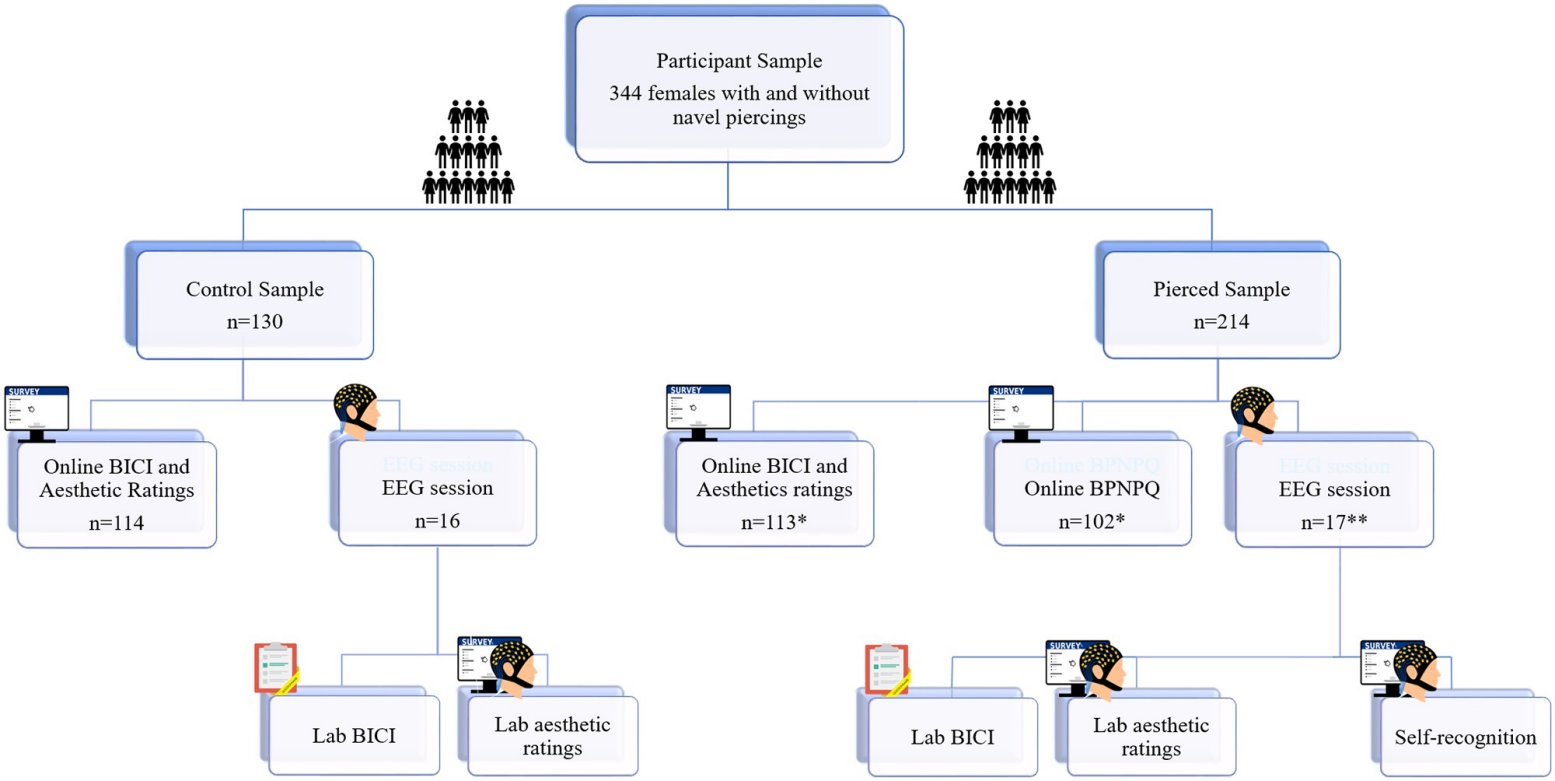
Overview of participant samples and measures. Note that not all participants contributed to all tasks (see also Results). * Of these, n = 18 completed both Online BICI and Aesthetic ratings and Online BPNPQ. ** EEG data was recorded from n = 16 only; one additional participant contributed behavioural responses only.

Not all participants contributed to all tasks (for details see Fig 1 and Results).

The study complied with the ethical standards laid down in the 1964 Declaration of Helsinki and its later amendments and was approved by the University of Essex Faculty of Science and Health ethics subcommittee (approval number: HG1804). All participants gave their informed written consent to participate.

### 2.2. Stimuli and experimental tasks

#### 2.2.1. Body perception with and without Navel Piercings Questionnaire

The Body Perception with and without Navel Piercings Questionnaire (BPNPQ) was newly designed for this study because no existing questionnaire focuses on navel piercings / the abdominal region. The BPNPQ contains 47 questions in three parts (see https://osf.io/g7s8c). The first part of the BPNPQ probed participants on the duration and frequency of wearing their navel piercing, reason(s) for choosing to get a navel piercing, and changes in clothing style as a result of the navel piercing.

The second part of the BPNPQ measured changes in body image and self perception as a result of the navel piercing. Nine features of body image and self perception (including body ownership and agency aspects of self-awareness) were assessed: feelings of being yourself (body ownership), control of body and actions (agency), satisfaction with shape and with size of body, satisfaction with sight of stomach, perceived attractiveness to others, perceived success in life, comfort with seeing one’s own body and with others seeing one’s body. For each feature, we asked participants (in three separate blocks) to rate their experience at three different time points: before they got their navel piercing, after they got their navel piercing, and if they imagined temporarily removing their piercing (e.g., “Before you got your navel piercing (vs. after you got your navel piercing, vs. if you were to temporarily remove your navel piercing), how much did / would you feel like yourself?”). Responses were made on a visual analogue scale (VAS) ranging from 0 (e.g. “not at all myself”) to 100 (e.g. “completely myself”), where the cursor’s starting point was centred (at 50).

In the third part of the BPNPQ, participants were asked to indicate on a 7-point Likert scale from strongly disagree to strongly agree whether they agreed with each of the statements “I hated my stomach until I pierced my belly button” and “Fortuitous events have occurred since I pierced my navel”.

#### 2.2.2. Body Image Concern Inventory (BICI)

The BICI is a 19-item questionnaire used to measure dysmorphic appearance concerns and related behaviours [35], which are common in individuals with eating and body dysmorphic disorders. Participants are asked to select how often they experience the described feeling (e.g., “I am ashamed of some part of my body”) or perform the described behaviour (e.g., “I spend a significant amount of time checking my appearance in the mirror”) on a 5-point likert scale from 1 (“never”) to 5 (“always”). BICI has good internal consistency, with a Cronbach’s alpha of 0.93 and item total correlations averaging 0.62. The total score ranges from 19 to 95, with higher scores indicating greater body image concern as reflected in negative feelings towards one’s body, social avoidance, and checking and camouflaging behaviours. Scores above 72 or 55 have been deemed as clinically concerning by Littleton et al., [35] and Schulte-van Maaren et al., [52], respectively.

#### 2.2.3. Aesthetic ratings task

**Fig 2:**
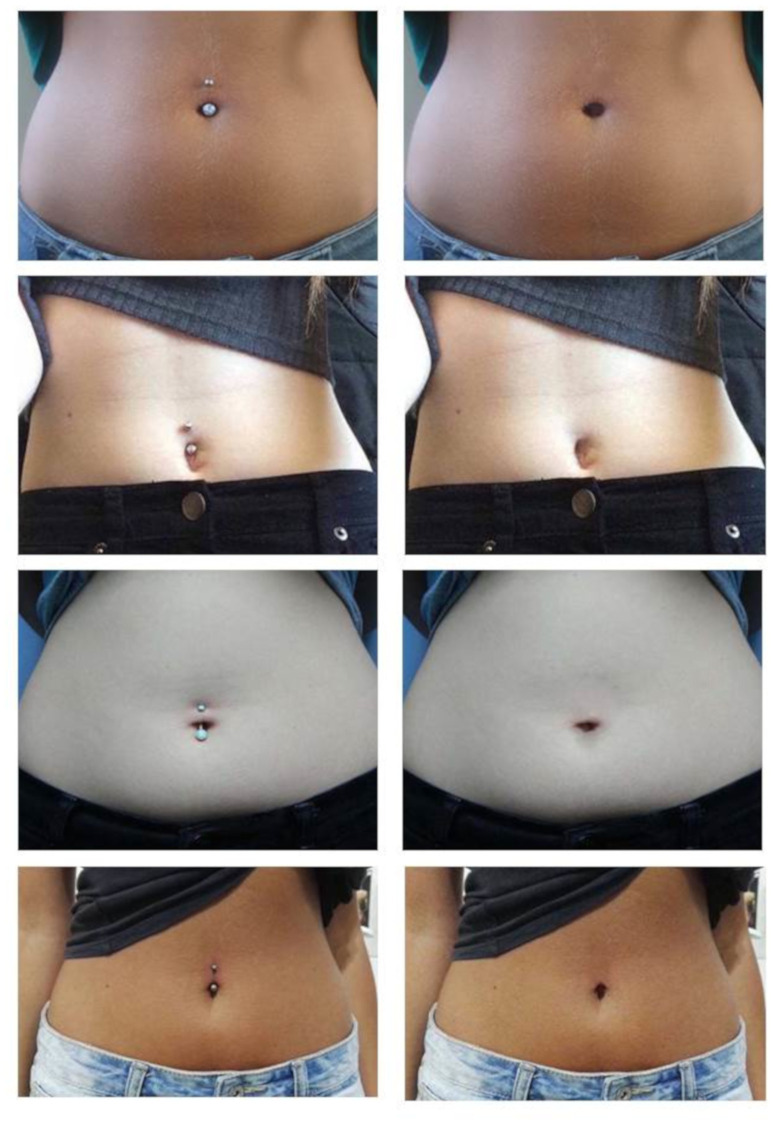
Examples of images depicting women’s abdomens with (left panel) and without (right panel) navel piercings.

This task involved viewing a series of images of women’s abdomens with and without navel piercings (for examples see Fig 2). In their own time, participants had to indicate on a visual analogue scale (VAS) from 0 (“not at all appealing”) to 100 (“extremely appealing”) how appealing they found the displayed person’s body.

A total of 28 bodies were shown (14 images of bodies with navel piercings and 14 images of the same bodies without navel piercings, presented in pseudorandom order to avoid direct repetitions of the same body with and without piercing). Images were obtained from the world wide web, and navel piercings were removed with graphics software to create the unpierced bodies.

The aesthetic ratings task was either completed online as described above, or was completed as part of the EEG session, embedded in a larger set of 190 passively viewed images of women’s abdomens. A larger set was necessary to accrue sufficient trials for visual ERPs. During the EEG session, each trial began with a white screen presented for a random interval between 600 and 1000 ms, followed by an image presented for 1200 ms in the centre of the white screen. The total stimulus set consisted of 27 images of bodies with navel piercings and 27 images of the same bodies without navel piercings, each presented three times. For 28 images (14 with and 14 without piercings) from this set aesthetic ratings were obtained, and these were presented a fourth time, and were then presented together with the VAS, which participants completed in their own time. These 28 passive viewing-plus-rating trials were interspersed among the passive viewing-only trials, leading to a total of 190 trials (95 showing bodies with and 95 showing bodies without navel piercings). The visual angles of the images were 15° (vertical) and between 13° and 29° (horizontal), depending on the image.

Participants completed the BICI and the aesthetic ratings task either online or as part of the EEG session in person at the University of Essex Centre for Brain Science. None of the EEG participants had participated in the aesthetic ratings task online. The BPNPQ was always completed online.

#### 2.2.4. Self-recognition task

This task was completed by pierced participants who took part in the EEG session only, and required speeded responses to images of women’s abdomens, identifying them as either their own or another woman’s abdomen. Participants were asked to provide two images of their abdomen, one with their navel piercing in, and the second with the navel piercing out. The second image was then digitally edited with Adobe Photoshop and Photopea to remove the holes created by the piercing, to provide an image of the navel as it would have been prior to any piercing. The two self-images were each presented 40 times, accompanied by four of the images of bodies (with and without piercings) from the larger set of 27 (described in section 2.2.3.), which were chosen to match the participant’s images in mean luminosity to control effects on ERPs from low-level visual differences between images. Each of these eight different images of other bodies was presented ten times. The visual angles of the images in the self-recognition task were 10° vertical and between 9° and 14° horizontal, depending on the image.

Each trial began with a 500-ms presentation of a black fixation cross on a grey background, followed by the 250-ms presentation of an image in the centre of the grey screen, followed by a 1000-ms response interval during which a blank grey screen was presented. Participants were required to indicate, by pressing 1 or 2 on the keypad with one of two fingers of their right hand, whether the image presented was of their own or another woman’s abdomen as quickly and accurately as possible during this response interval. The inter-trial interval, showing a blank grey screen, was a randomly chosen interval between 300 and 600 ms. There were 160 trials altogether (80 self-images, 80 other-images), and cumulative performance feedback on overall speed and accuracy was provided every 40 trials.

### 2.3. EEG procedure

All participants who participated in the EEG session were given a paper version of the BICI to complete. Participants were then fitted with the scalp EEG cap and facial electrooculography (EOG) electrodes. Participants with navel piercings completed the aesthetic ratings task, followed by the self-recognition task. Control participants underwent the same procedure, but were only asked to complete the aesthetic ratings task.

#### 2.3.1. Electrophysiological recordings

Recording and offline analysis of EEG and EOG data was done with Neuroscan Synamps 2 system and Scan 4.5 software (Compumedics, Melbourne, Australia). During the task(s), EEG activity was recorded continuously from 64 scalp electrodes placed according to the international 10–10 system (EASYCAP GmbH, Herrsching, Germany), referenced to the left earlobe. Horizontal and vertical EOG was recorded from electrodes placed above and below the left eye (vEOG) and beside the outer canthi of both eyes (hEOG). Impedances were kept below 35kΩ. EEG and EOG were amplified, band-pass filtered at 0.05–100 Hz, and digitised at 2000 Hz.

#### 2.3.2. EEG / EOG pre-processing and ERP analysis

Offline, the EEG / EOG signals were digitally filtered using a 30-Hz low-pass filter (12 dB slope) and re-referenced to the average of all scalp electrodes. Eye blinks were removed using Scan 4.5’s ocular artifact removal method based on Semlitsch et al., [53]. Data were epoched from 100 ms before stimulus onset to 800 ms after stimulus onset. Epochs with artifacts (events exceeding +/-100μV for 300 ms after stimulus onset relative to the 100−ms pre−stimulus baseline) were removed from the dataset. The remaining epochs were averaged for each stimulus type (with piercing vs. without piercing in the aesthetic ratings task; self with piercing vs. self without piercing vs. other with piercing vs. other without piercing in the self-recognition task).

For P1, N1 and N250 components we extracted peak amplitudes centred around the components peaks obtained from the grand-averaged waveforms collapsed across all conditions in each task. We extracted positive peak amplitudes between 90 and 130 ms (P1, aesthetic ratings task) or between 80 and 125 ms (P1, self-recognition task), negative peak amplitudes between 130 and 175 ms (N1, both tasks), and negative peak amplitudes between 240 and 280 ms [N250, self-recognition task; see e.g., 47] from posterior electrodes P7/8, P5/6, PO7/8, PO5/6 and O1/2 separately for each participant. Electrode selection was determined on the basis of visual inspection to identify sites showing maximal ERP component sizes in the grand-averaged data collapsed over all conditions separately for each task [see 41]. For LPP, we extracted mean amplitudes between 300 and 700 ms for fronto-central electrodes F5/6, F3/4, F1/2, FC5/6, FC3/4 and FC1/2 separately for each participant. LPP time window and electrode selection were determined on the basis of visual inspection to identify sites showing a positive-going wave (the LPP) in the grand-averaged data collapsed over all conditions separately for each task [see 54, 55].

To obtain P1-N1 peak-to-peak amplitude values, we subtracted N1 peak amplitudes from P1 peak amplitudes for each participant and electrode. For the aesthetic ratings task, P1-N1 peak-to-peak and LPP mean amplitudes were analysed with repeated measures ANOVAs for the factors piercing (with piercing vs. without piercing), hemisphere (left vs. right) and electrode (P7/8 vs. P5/6 vs. PO7/8 vs. PO5/6 vs. O1/2 for P1-N1, and F5/6 vs. F3/4 vs. F1/2 vs. FC5/6 vs. FC3/4 vs. FC1/2 for LPP), and the between-subject factor group (pierced participants vs. controls). For the self-recognition task, P1-N1 peak-to-peak, N250 peak amplitudes and LPP mean amplitudes were analysed with repeated measures ANOVAs for the factors identity (self vs. other), piercing (with piercing vs. without piercing), hemisphere (left vs. right) and electrode (P7/8 vs. P5/6 vs. PO7/8 vs. PO5/6 vs. O1/2). Significant interactions between piercing and group (aesthetic ratings task) and between identity and piercing (self-recognition task) were followed up with pairwise comparisons of the relevant estimated marginal means. Greenhouse-Geisser corrected statistics are reported where assumptions of sphericity were not met. All reported power calculations were completed post-hoc.

## 3. Results

### 3.1. Body perception with and without navel piercings

A total of 102 participants with navel piercings (mean age: 25 years, SD: 6.9, range: 19–55) completed the BPNPQ. Ninety-seven of them (95.1%) reported having additional piercings, and of those, 51.5% had additional piercings apart from ear piercings. 59.8% of participants indicated that they have had their navel piercing for 5 or more years (the longest time was 24 years), 11.8% had it for 3 to 5 years, 21.6% for 1 to 3 years and 6.9% for less than a year (the shortest time was 1 month).

Table 1 provides the frequency of scores for motivations chosen to get a navel piercing. The most common reason for obtaining a navel piercing was to ‘increase physical attractiveness’, selected by around half of all respondents (53/102) as one of their motivations. Of all the 194 indicated motivations collected across respondents, 27% were to increase physical attractiveness, while the next most common reasons (rebelliousness, independence, control over body) each composed around 12% of all motivations. Reasons related to body image (increasing physical attractiveness (27%), control over one’s own body (12%), and hiding a flaw (3%)) together made up 42% of all motivations. Overall, these results suggest that navel piercings in women are foremost intrinsically linked with body image, and less so with individuality or rebelliousness.

**Table 1:** List of motivations ranked from the most common to the least common. *Other refers to answer akin to ‘simply wanted one’ or ‘impulsive decision’. F means the total amount of reasons selected by the participants, as each participant could select more than one answer.

Motivation	F (194)	Percentage
Increased physical attractiveness	53	27.3%
Rebelliousness	25	12.9%
Independence	25	12.9%
Control over body	23	11.9%
Need for uniqueness	20	10.3%
Positive attention from peers	17	8.8%
Attractiveness of piercing	13	6.7%
*Other	9	4.6%
Hide a flaw	6	3.1%
Peer pressure	3	1.5%
Cultural tradition	0	0%

The BPNPQ also indicated that respondents noticed a little change in their clothing style since their navel piercing (e.g., wearing more crop tops) (average score of 27.9/100, where 0 was none at all, 50 was a moderate amount and 100 was a lot).

Reliability analyses were carried out on the nine items (body image and self perception questions) from each block (before piercing, since piercing, and imagined temporary removal of piercing) of the second part of the BPNPQ. This indicated good to excellent internal consistency throughout. Coefficient alpha for the before piercing items was .92 [95% confidence interval: .90 to .94; 56, 57]. For the since piercing items coefficient alpha was .92 (95% confidence interval: .89 to .94), and for items in imagined temporary removal of piercing coefficient alpha was .92 (95% confidence interval: .90 to .95). Item total correlations ranged from .52 to .87 for before piercing items, from .50 to .86 for since piercing items, and from .52 to .86 for items in imagined temporary removal of piercing. Lower item total correlations were obtained for success in life (.50 to .57), followed by self-awareness (agency, body ownership) items (.52 to .63), and highest item total correlations were obtained for body image items (.71 to .87).

The VAS ratings of the three blocks of nine questions were then analysed with a repeated measures analysis of variance (ANOVA) comparing the effect of the time (before piercing, since piercing, and imagined temporary removal of piercing) and its interaction with each of the nine features that were queried. There was a significant main effect of time, F(2,202) = 31.98, p < .001, ƞ_p_^2^ = .24, 1-β = 1.0, a significant main effect of feature, F(8,808) = 33.16, p < .001, ƞ_p_^2^ = .25, 1-β = 1.0, as well as a significant interaction between time and feature, F(16,1616) = 12.08, p < .001, ƞ_p_^2^ = .11, 1-β = 1.0). Pairwise comparisons of the estimated marginal means for each level of time suggest that getting a navel piercing has positive effects on an individual’s body image and self perception, which significantly improves (M = 69.5, SEM = 1.8) relative to before the navel piercing (M = 59.6, SEM = 2.2; pairwise comparisons of the estimated marginal means: p < .001). Body image and self perception diminish when imagining the temporary removal of the piercing (M = 58.3, SEM = 2.3; pairwise comparisons: p < .001). However, this pattern of change further depended on the feature of body perception queried. Table 2 provides a summary of the pairwise comparisons of the estimated marginal means for each of the nine features across time.

**Table 2:** Mean VAS ratings (SEMs) for each of the nine body image and self perception questions across the three conditions of time. Single asterisks (*) indicate significant differences (t(101)≥2.83; p≤.006, dz≥1.74, 1-β = 1.0) from body perception since getting pierced (grey column) in Bonferroni-corrected pairwise comparisons of estimated marginal means; double asterisks (**) indicate p≤.001.

Question	Time		
	Before getting navel piercing	Since getting navel piercing	Imagined temporary removal of navel piercing
1. How much did you feel like yourself?	75.0 (2.4)**	81.5 (1.8)	58.9 (3.3)**
2. How much did you feel in control of your body and actions?	76.3 (2.6)**	84.3 (1.9)	74.2 (2.9)**
3. How satisfied were you with the shape of your body?	53.7 (3.0)**	64.5 (2.6)	59.5 (3.0)*
4. How satisfied were you with the size of your body?	58.2 (3.2)	61.5 (2.8)	59.3 (2.9)
5. How satisfied were you with the sight of your stomach?	54.7 (3.1)**	68.6 (2.5)	46.0 (3.2)**
6. How attractive did you think you were to others?	50.6 (2.5)**	64.2 (1.8)	56.5 (2.4)**
7. How successful did you feel in life?	60.9 (2.4)**	67.2 (2.0)	67.0 (2.3)
8. How comfortable did you feel when you saw your body?	56.3 (2.8)**	67.8 (2.4)	51.8 (3.0)**
9. How comfortable did you feel when others saw your body?	50.8 (2.9)**	65.4 (2.5)	51.8 (2.9)**

Most features (1, 2, 3, 5, 6, 8, and 9) significantly differed between before-piercing and since-piercing conditions, and between since-piercing and imagined-removal conditions. For some of the features (1, 5, and 8), imagining temporary removal of the piercing even led to more negative body and self perceptions than those stated before getting the navel piercing. This suggests a strong integration of their navel piercing into individuals’ bodily self image, such that the continued presence of the piercing is seen as necessary for feeling oneself and for being satisfied and comfortable with the sight of one’s body. Navel piercings selectively improved individuals’ satisfaction with their body shape, but not with their body size, since satisfaction with body shape (feature 3) changed over time, but satisfaction with body size (feature 4) showed no changes. Finally, getting a navel piercing increased how successful an individual felt in life (feature 7), but this feeling did not depend on the continued presence of the piercing, and thus remained elevated even with imagined temporary removal.

Of the 101 participants who completed the two additional statements, around one third (34.7%) agreed with the statement “I hated my stomach until I pierced my belly button”, and a minority (13.9%) agreed with the statement “Fortuitous events have occurred since I pierced my navel”. The majority of respondents disagreed with the two statements (50.5% and 48.5%, respectively), and the remainder neither agreed nor disagreed. This suggests that around a third of women were able to overcome negative feelings about their abdominal region as a result of piercing their navel. However, only a few women also felt that their piercing led to other, fortuitous events in their lives.

### 3.2. Body dysmorphic concerns

A total of 260 participants, 130 with and 130 without navel piercings, completed the BICI. 227 of these participants completed the BICI online and 33 completed the BICI as part of the EEG session. The average age of participants was 25.8 years in the pierced group, and 25.6 years in the control group.

**Fig 3:**
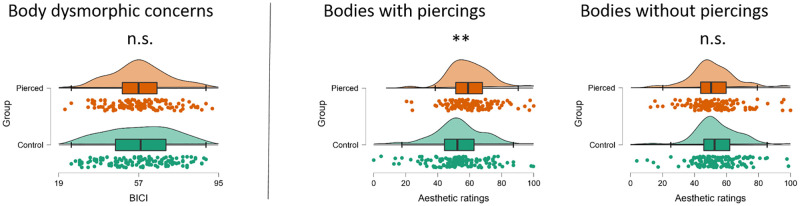
Raincloud plots of BICI scores (left panel) and aesthetic ratings of bodies with and without navel piercings (right two panels) in participants with (orange) and without (turquoise) navel piercings. Each circle represents one participant, n.s. denotes a non-significant group difference, ** denotes p < .001.

The average BICI score was 57.5 for the pierced group and 57.5 for the control group (sub-clinical according to Littleton et al., [35], but close to the clinical cut-off of 55 according to Schulte-van Maaren et al., [52]). An independent samples t-test confirmed that there was no significant difference in dysmorphic appearance concerns between individuals with and without navel piercings, t(258) = .008, p = .993, d < .01 (see Table 3 and Fig 3). In the pierced sample, the lowest score obtained was 22 and the highest 92, and 19 participants scored in the clinical range of 72 and above [35]. In the control sample, the lowest and highest score obtained were 25 and 89, and 27 participants scored in the clinical range. These results suggest that there is no evidence that women with navel piercings have more body image concerns compared to women without navel piercings.

**Table 3:** Sample characteristics, BICI scores and aesthetic ratings. Tests of comparisons used chi square and independent-sample t-tests. Asterisks denote significant differences between groups.

	Pierced group (n = 130)	Control group (n = 130)	Tests of Comparison
**Age** Mean (SD)	25.8 (6.4)	25.6 (7.0)	t (258) = 0.212, p = 0.833
**Medical history** N (%):			
Eating Disorders	17 (13.1)	11 (8.5)	x^2^(1) = 1.441, p = 0.230
Body Dysmorphic Disorder	4 (3.1)	4 (3.1)	x^2^(1)<0.001, p = 1.000
Other conditions	63 (48.5)	54 (41.5)	x^2^(1) = 1.259, p = 0.262
**BICI** Mean (SD)	57.5 (13.7)	57.5 (16.0)	t(258) = 0.008, p = 0.993
**Aesthetic ratings of bodies with navel piercings** Mean (SD)	60.3 (13.6)	52.9 (17.1)	t(258) = 3.834, p<0.001, d = .48 **
**Aesthetic ratings of bodies without navel piercings** Mean (SD)	52.0 (15.2)	53.8 (14.8)	t(258) = -0.973, p = 0.331

Self-reported medical history was also similar across pierced and control groups (see Table 3). There were 17 pierced participants who have or have had an eating disorder (vs. 11 in the control group), 4 body dysmorphic disorder (vs. 4 in the control group), and 63 other mental health conditions (vs. 54 in the control group). These results suggest that incidences of disorders characterised by body image disturbances were also no more prevalent in women with navel piercings than in those without.

### 3.3. Aesthetic ratings of bodies with and without navel piercings

The same samples as described in 3.2. above participated in the aesthetic ratings task, and their mean aesthetic ratings of bodies with and without navel piercings are displayed in Table 3 and Fig 3. A repeated-measures ANOVA revealed no main effect of sample (pierced vs. control) on overall aesthetic ratings, F(1,258) = 2.7, p = .118, ƞ_p_^2^ = .01, 1-β = .87). Participants with navel piercings gave similar aesthetic ratings (56.2) as participants without navel piercings (53.4). There was a significant main effect of image (bodies with vs. without navel piercings), F (1,258) = 29.1, p < .001, ƞ_p_^2^ = .10, 1-β = 1.0, as well as a significant interaction between sample and image, F (1,258) = 45.5, p < .001, ƞ_p_^2^ = .15, 1-β = 1.0). Participants with navel piercings rated images of bodies with navel piercings as significantly more appealing than images of bodies without navel piercings (60.3 vs. 52.0). In contrast, control participants rated images of bodies with and without navel piercings as similarly appealing (52.9 vs. 53.8). Independent t-tests confirmed that the groups differed specifically in their aesthetic appreciation of bodies with navel piercings (see Table 3 and Fig 3).

### 3.4. Visual ERPs to bodies in aesthetic ratings and self-recognition tasks

A total of 17 participants with navel piercings (mean age: 22.1 years; 15 right-handed) and 16 control participants without navel piercings (mean age: 22.2 years; 15 right-handed) completed the aesthetic ratings and self-recognition tasks as part of the EEG session. Since we were unable to collect EEG responses from one (pierced) participant on the day, this person only contributed behavioural responses. None of the EEG participants had been diagnosed with an eating disorder or body dysmorphic disorder. However, five pierced participants had been diagnosed with another condition (anxiety, cyclothymia, depression, insomnia), and three control participants had been diagnosed with another condition (adjustment disorder, anxiety, depression).

**Fig 4:**
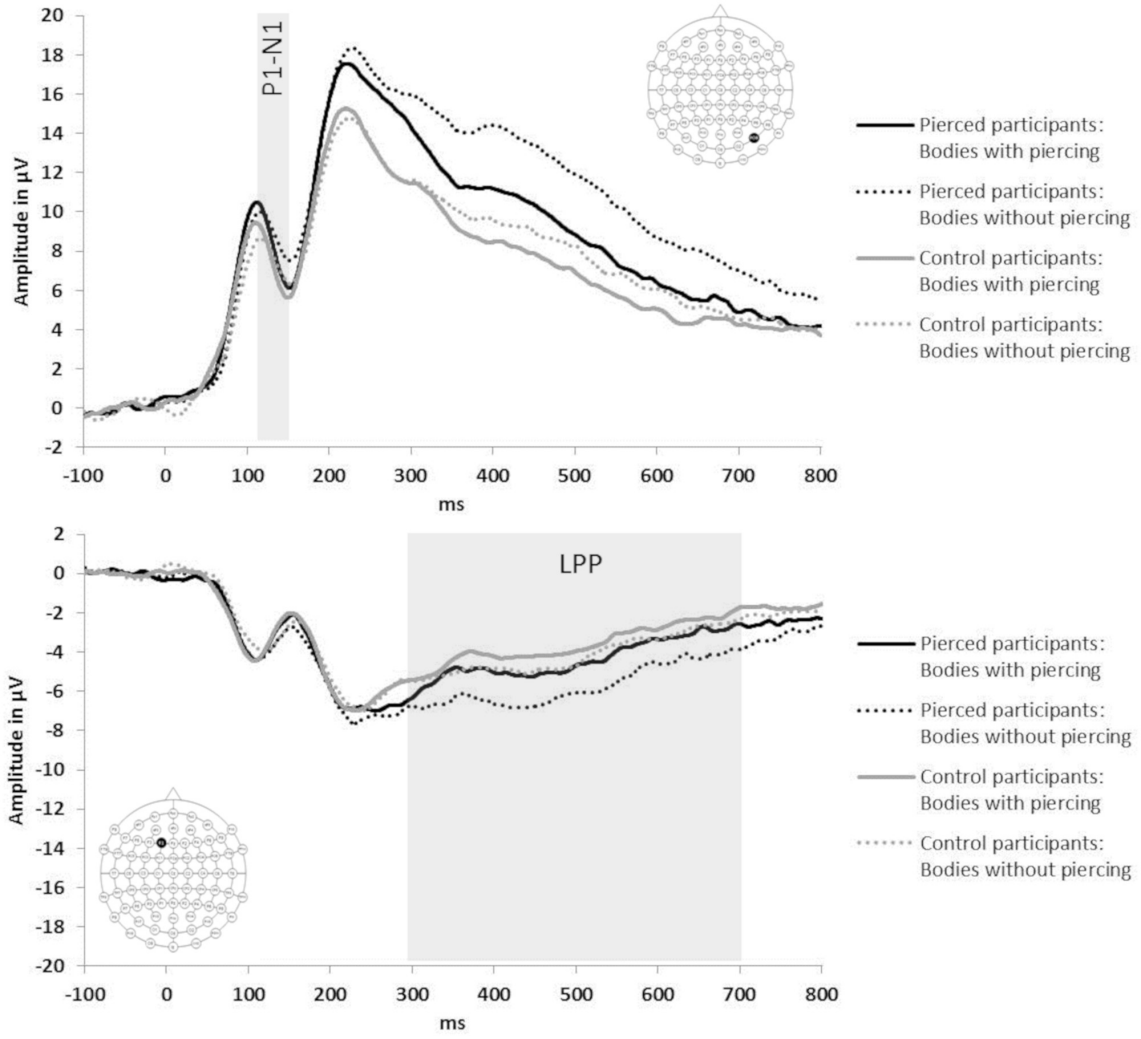
Visual ERPs to images of other women’s abdomens with (solid waveforms) and without (dotted waveforms) navel piercings obtained during the aesthetic ratings task from participants with (black waveforms) and without (grey waveforms) navel piercings. Top panel shows right-hemispheric posterior electrode PO8; bottom panel shows left frontal electrode F1 (see insets for electrode locations).

**Fig 5:**
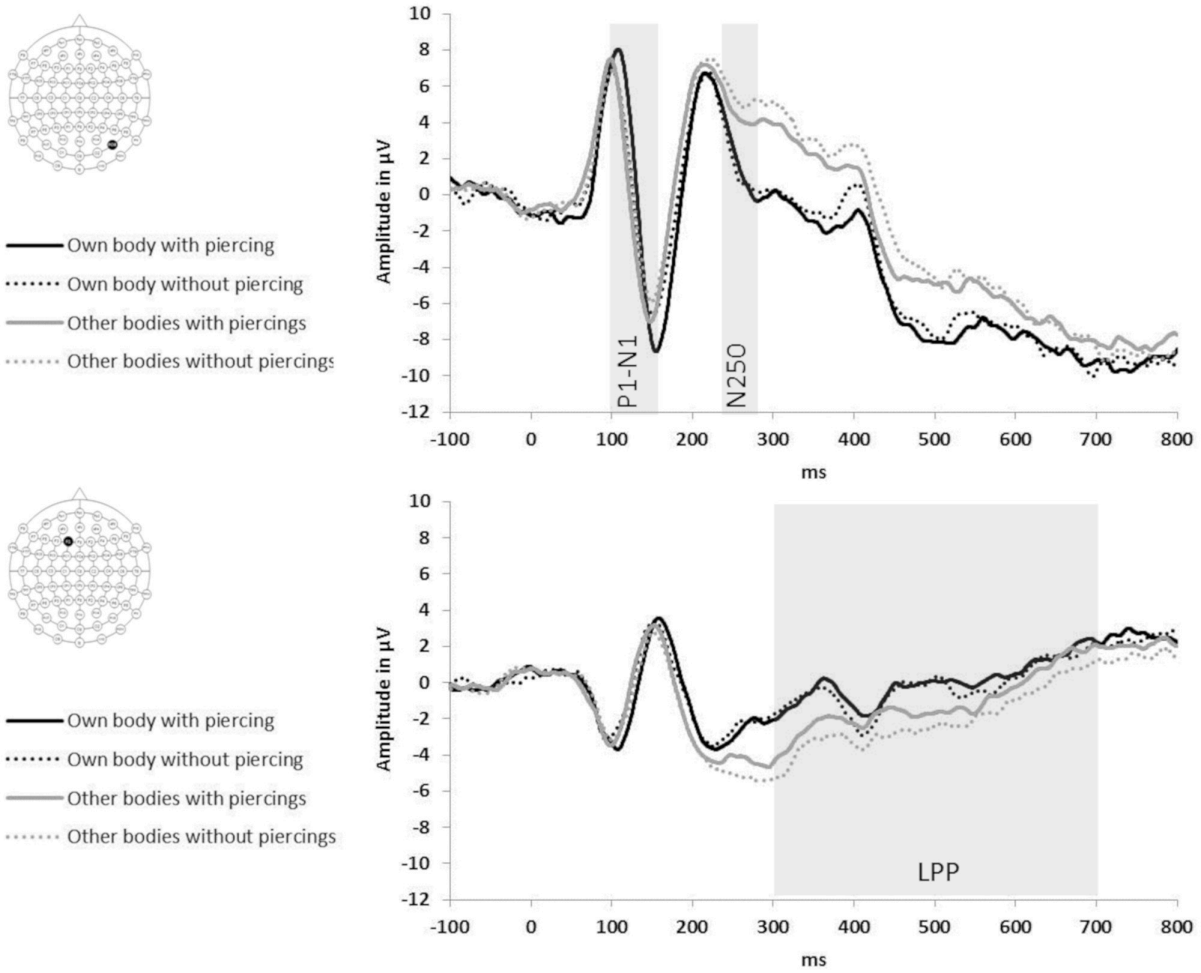
Visual ERPs, obtained during the self-recognition task, to images of participants’ own abdomen (black waveforms) and other women’s abdomens (grey waveforms) depicted with (solid waveforms) and without (dotted waveforms) navel piercings. Top panel shows right-hemispheric posterior electrode PO8; bottom panel shows left frontal electrode F1 (see insets for electrode locations).

For the aesthetic ratings task (see Fig 4), bodies with navel piercings gave rise to larger posterior P1-N1 peak-to-peak amplitudes, as well as to a larger LPP over frontal electrode sites, than bodies without piercings. These differences were more pronounced in the group with navel piercings than in the control group. For the self-recognition task (see Fig 5), a similar pattern emerged. Bodies with piercings gave rise to larger posterior P1-N1 peak-to-peak amplitudes, larger posterior N250 amplitudes, as well as to a larger LPP at frontal electrode sites, than bodies without piercings. For images depicting participants’ own body, there were also larger posterior P1-N1 peak-to-peak amplitudes, especially in the right hemisphere, larger N250 amplitudes, and a larger frontal LPP, compared to images depicting others’ bodies.

#### 3.4.1. ERPs related to visual body-structural encoding (P1-N1 complex)

In the *aesthetic ratings task*, repeated measures ANOVAs of P1-N1 peak-to-peak amplitudes showed a main effect of piercing, F(1,30) = 42.3, p < .001, η_p_^2^ = .59, 1-β = 1.0, Peak-to-peak amplitudes were larger for bodies with piercings than without piercings for both groups of pierced and control participants (piercing x group: F(1,30)<1, p = .731, η_p_^2^ < .01, 1-β < .11). The interaction of the effect of piercing with hemisphere and group just missed significance, F(1,30) = 3.9, p = .057, η_p_^2^ = .12, 1-β = .98. Pairwise comparisons of the estimated marginal means of bodies with and without piercings at each hemisphere and for each group showed that the attention-enhancing effect of piercings at P1-N1 was greatest at right-hemisphere sites for pierced participants (Δ1.60μV; F(1,30) = 26.2, p < .001, η_p_^2^ = .47, 1-β = 1.0) and smallest at left-hemisphere sites for pierced participants (Δ0.69μV; F(1,30) = 6.8, p = .014, η_p_^2^ = .19, 1-β>.99). For control participants the effect of piercing on P1-N1 was intermediate and similar across hemispheres (right hemisphere: Δ1.02μV; F(1,30) = 10.6, p = .003, η_p_^2^ = .26, 1-β>.99; left hemisphere: Δ1.04μV; F(1,30) = 15.6, p < .001, η_p_^2^ = .34, 1-β = 1.0). In summary, during the aesthetic rating of bodies, the presence of navel piercings enhances visual body recognition in all observers at early cortical stages, and these effects are strongly lateralised to the right hemisphere in participants who themselves have a navel piercing.

The main effect of piercing on P1-N1 peak-to-peak amplitudes was replicated in the *self-recognition task*, F(1,15) = 14.6, p = .002, η_p_^2^ = .49, 1-β = 1.0. Repeated measures ANOVAs of P1-N1 peak-to-peak amplitudes also showed a main effect of identity, F(1,15) = 17.2, p = .001, η_p_^2^ = .54, 1-β = 1.0. Peak-to-peak amplitudes were larger for bodies with piercings (13.8μV) than bodies without piercings (12.6μV), and larger for images of the self (14.4μV) than of other women’s bodies (12μV). There were significant interactions between identity and hemisphere, F(1,15) = 6.5, p = .022, η_p_^2^ = .30, 1-β>.99, between piercing and electrode, F(4,60) = 6.4, p = .002, η_p_^2^ = .30, 1-β>.99, and a four-way interaction between identity, piercing, hemisphere and electrode, F(4,60) = 3.48, p = .030, η_p_^2^ = .19, 1-β>.99. Pairwise comparisons of the estimated marginal means of self and other at each level of piercing, hemisphere and electrode showed that the P1-N1 enhancement for self-body images compared to other-body images *with* piercings was restricted to right-hemisphere electrodes (all F(1,15)≥5.3, p≤.036, η_p_^2^≥.26, 1-β>.99) and to left-hemisphere electrode O1 (F(1,15) = 4.7, p = .047, η_p_^2^ = .24, 1-β>.99; all other left-hemisphere electrodes F(1,15)≤3.6, p≥.077, η_p_^2^≤.19, 1-β>.99). In contrast, P1-N1 enhancement for self-body images *without* piercings was present for all left- and right-hemisphere electrodes (all F(1,15)≥6.6, p≤.022, η_p_^2^≥.30, 1-β>.99) except left-hemisphere P7 (F(1,15) = 2.3, p = .149, η_p_^2^ = .13, 1-β = .96).

In sum, early cortical body-structural encoding (as indexed by P1-N1 amplitudes) was affected by the presence of navel piercings (greater attentional engagement with piercing) in both tasks. ERPs also showed an enhancement for images of one’s own than other women’s bodies (self-advantage) in the self-recognition task. The self-advantage was bilateral when viewing bodies *without* navel piercings, but largely restricted to the right hemisphere for bodies *with* navel piercings.

#### 3.4.2. ERPs related to identity recognition (N250)

In the *self-recognition task*, repeated measures ANOVAs of N250 peak amplitudes showed a main effect of identity only, F(1,15) = 50.4, p < .001, η_p_^2^ = .77, 1-β = 1.0. N250 peak amplitudes were larger for images of the self (-0.5μV) than of other women’s bodies (2.9μV), but this was independent of the presence of navel piercings (all interactions involving identity and piercing: F≤3.25, p≥.091, η_p_^2^≤.18, 1-β≤.99).

This confirms that, just like for faces, identification of bodies is associated with electrophysiological changes in the N250 time window, but there are no further enhancements from the presence of navel piercings.

#### 3.4.3. ERPs related to emotional-motivational processes (LPP)

Repeated measures ANOVAs of mean LPP amplitudes at frontal electrodes in the *aesthetic ratings task* showed a main effect of piercing, F(1,30) = 54.9, p < .001, η_p_^2^ = .65, 1-β = 1.0, which interacted with group, F(1,30) = 9.0, p = .005, η_p_^2^ = .23, 1-β>.99. Waveforms were more positive for bodies with piercings (-3.3μV) than bodies without piercings (-4.1μV), and this difference was twice as large in the participants with navel piercings (Δ1.1μV) than in the control group (Δ0.5μV) (both F(1,30)≥9.8, p≤.004, η_p_^2^≥.25, 1-β≥.99).

The main effect of piercing at LPP was replicated in the *self-recognition task*, F(1,15) = 5.8, p = .030, η_p_^2^ = .28, 1-β>.99. Repeated measures ANOVAs of mean LPP amplitudes at frontal electrodes also showed a main effect of identity, F(1,15) = 10.6, p = .005, η_p_^2^ = .41, 1-β = 1.0). LPP amplitudes were more positive for bodies with piercings (0.1μV) than bodies without piercings (-0.3μV), and more positive for images of the self (0.4μV) than of others’ bodies (-0.6μV). There were no significant interactions between identity and piercing (all interactions involving identity and piercing F≤1.11, p≥.309, η_p_^2^≤.07, 1-β≤.69).

To summarise, during both self-recognition and aesthetic rating of bodies, the presence of navel piercings enhances frontal emotional-motivational processing at late, cognitive stages in all observers, but these effects are more pronounced in women who themselves have a navel piercing. Viewing images of one’s own body is also associated with electrophysiological changes in this time window (leading to enhanced emotional processing), but there are no further enhancements from the presence of navel piercings.

### 3.5. Speed of self-recognition with and without navel piercings

Only the pierced sample who completed the EEG session (N = 17, mean age: 22.14 years; 15 right-handed) participated in this task, which was part of the EEG sessions.

**Fig 6:**
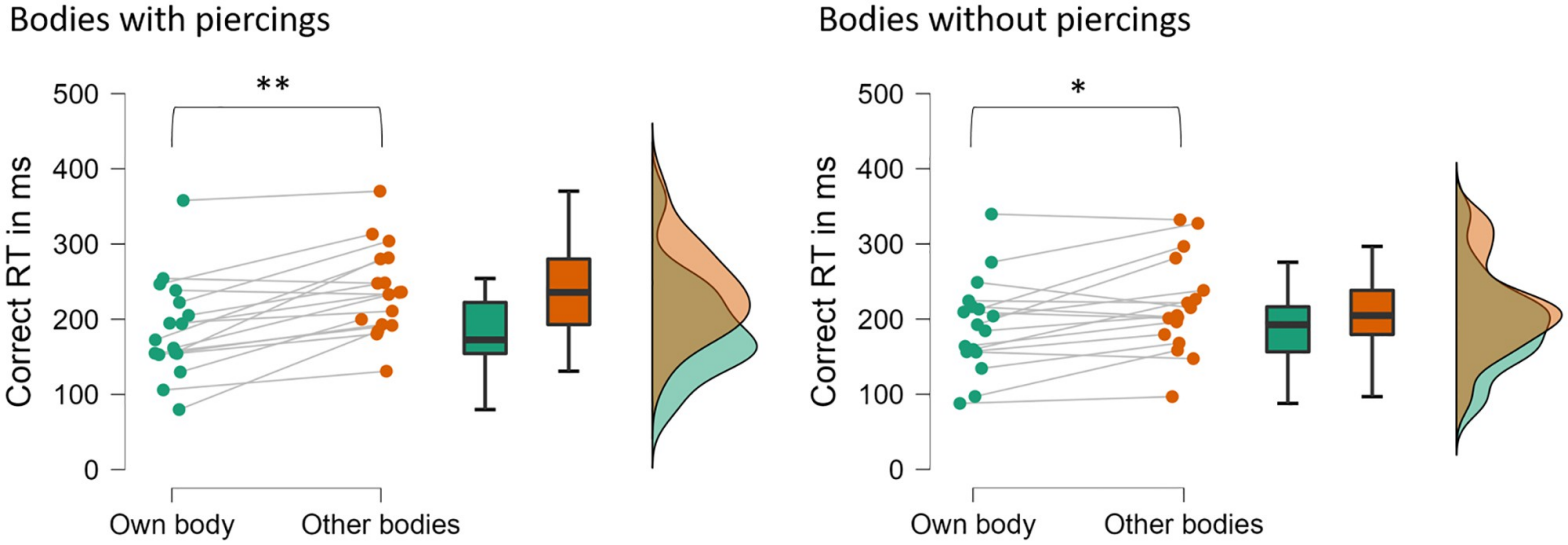
Raincloud plots of correct RTs to own (turquoise) and strangers’ bodies (orange) with (left panel) and without (right panel) navel piercings. Each circle represents one participant, * denotes p < .05, ** denotes p < .001.

Missed and incorrect responses were infrequent (<3% of trials on average). Correct RTs from the remaining trials (see Fig 6) were entered into a repeated measures ANOVA with the factors identity (self vs. other) and piercing (with piercing vs. without piercing). There was a significant main effect of identity, F(1,16) = 23.46, p < .001, η_p_^2^ = .60, 1-β = 1.0, and a significant interaction between identity and piercing, F(1,16) = 7.22, p = .016, η_p_^2^ = .31, 1-β>.99. Responses were faster for images of the self (189.5 ms) than of others’ bodies (227.5 ms), and this difference was twice as large for bodies with piercings (50.6ms; F(1,16) = 7.22, p < .001, η_p_^2^ = .64, 1-β = 1.0) than for bodies without piercings (25.3ms; F(1,16) = 8.36, p = .011, η_p_^2^ = .34, 1-β = 1.0).

In other words, there was a self-advantage on the speed of responding to images of women’s abdomens. This self-advantage was larger for bodies with navel piercings than for bodies without navel piercings, suggesting that individuals have integrated their navel piercing into their visual bodily self image.

## 4. Discussion

Navel piercings are increasingly common among women. In the most recent representative survey of body piercings [7] one fifth (19.4%) of French metropolitan women (8.4% of men) had at least one piercing; and navel piercings were reported by one third of those women (one twentieth of men). The aim of the present study was to explore, using a variety of methods, the selective effects of navel piercings on body and self perception in women.

### 4.1. Motivations for navel piercings

In line with our hypothesis, we found that body image related reasons (increasing physical attractiveness, control over body, hiding a flaw) together made up 42% of all the motivations collected across respondents, with increasing physical attractiveness accounting for more than half of this figure (27%). Other common reasons (rebelliousness, independence, need for uniqueness) each composed only 10–13% of all motivations. This shows that navel piercings are far more strongly linked with body image than with other underlying motivations. These results are consistent with those of Kluger et al., [7], who found that embellishment was the most common reason for women obtaining a piercing (49% of women vs. 30% of men in their study), while men were most motivated by individuality [36% of men vs. 18% of women; see also 6]. Together with Kluger et al., [7] the present study differs from previous research on body piercings by explicitly distinguishing motivations by gender. This may explain why body image related reasons have thus far not been shown to dominate over other frequently cited motivations [e.g. 1, 5, 6, 13, 33].

### 4.2. Body image and self-perception in women with navel piercings

We hypothesised that, due to navel piercings’ place in history [1] and their association to aspects of self-image and identity [6, 7], the embellishment of the abdominal region would measurably improve women’s bodily self perception. In line with this, we found that getting a navel piercing increased individuals’ self-reported sense of identity, attractiveness, satisfaction and comfort with their visual body image following their navel piercing relative to before the piercing. We also show that, for most of those features, this improvement decreased again with imagined temporary removal of their piercing. In line with hypotheses, these results therefore suggest a strong integration of the navel piercing into individuals’ bodily self image, such that the continued presence of the piercing is seen as necessary by these women for feeling themselves, for feeling attractive, and for being satisfied or comfortable with the sight of their body. Free text comments further illustrate how navel piercings can become part of women’s self-identity and affect how they view their body, not just visually, but evaluatively and emotionally. One participant stated, “I had to take it out when I was pregnant and felt this was awful. I put it back in the day after I had my little boy.” Strong identification is in line with previous findings on body modifications in general. For example, Stirn & Hinz [58] showed that a majority of those with body modifications chose the statement “I am tattooed / body pierced” (83%) over the statement “I have a tattoo / body piercing” (17%).

In further confirmation of the positive effects on body image and self perception, around a third of women stated that they hated their stomach before their navel piercing. This suggests that embellishing an area of the body women feel negatively about [19] may allow at least some women to overcome these negative feelings toward their own body [see 25]. One respondent wrote, “I think it makes my stomach look pretty, something which I wouldn’t have said before I got it pierced. It makes me forget about my size and makes me focus on how much it glistens.” It should be noted that navel piercings selectively improved individuals’ satisfaction with their body shape, but not with their body size. While piercings cannot directly improve satisfaction with size, the above quote suggests that they may nevertheless provide a way to direct (negative) attention away from this aspect, and thus influence individuals’ feelings of attractiveness and comfortability with the size of their stomach [see also 25]. The findings further suggest that perceived size and perceived shape are somewhat independent aspects of (women’s) body image. Distinctions between size and shape may partly derive from independent processing of different visual aspects (size, shape, colour, position and motion) in separate perceptual modules [e.g., 59]. Further, the development of body shape perception (individual features and outer contours) depends less on visual experience than the perception of spacing between individual body features [60, 61].

Finally, the BPNPQ also indicated that the positive effects of navel piercings did not measurably extend beyond women’s physical body image. While getting a navel piercing increased how successful individuals felt in life, this feeling did not depend on the continued presence of the piercing. Success in life may have been assessed against external factors or the mere passage of time, independently of the piercing. The average age of getting a first piercing is around 19–20 years for women [7, 25, 58] and it is therefore likely that participants were assessing themselves at ages when they had naturally less vs. more personal and professional achievements in life.

Further, there was little self-reported change in clothing style and only a minority of women felt that fortuitous events occurred since their navel piercing. This suggests that, while individuals felt more attractive, this may not have resulted in the self-confidence [34] to measurably change their self-reported actions or behaviours, at least with regard to the behaviours probed by the BPNPQ.

In line with our hypothesis that navel piercings can improve body image in women, we found that women with navel piercings had no more body dysmorphic concerns [as assessed by BICI; 35] than women without navel piercings. Similarly, there are no differences in overall self-esteem [62] and mental health history [4] between adults of both genders with body modifications (tattoos and piercings) and those without. Claes et al., [25] even found that female eating disordered patients with piercings report less severe eating disorder symptoms than those without piercings. More than one third of these patients had navel piercings, and Claes et al., [25] refer to the possibility that such embellishments may reflect “positive body attitude, since many ED patients tend to focus on their ‘thick, fat, swollen’ belly” (p.17). Other studies of women, however, have suggested that those with more body piercings (and tattoos) may have a more negative body image in comparison to women with fewer such body modifications [63], and may also have a lower mental quality of life [7]. None of these studies specifically distinguished between piercing sites. Our study is thus the first to show that women with navel piercings have no more body image concerns, and no more incidences of disorders characterised by body image disturbances (e.g. eating disorders), than women without navel piercings.

We speculate that women with navel piercings scored similarly on the BICI as a result of having the navel piercing. This would be in line with the notion that body piercings may be an attempt to enhance one’s body image [7, 25, 58, see also 64]. The BPNPQ indicated that navel piercings can improve women’s perception of their own bodies, and this may subsequently result in less engagement in the excessive body surveillant and body avoidant thoughts and behaviours that are measured by the BICI. It may also be an expression of body care that can protect against self-harming behaviours, as advanced by Claes et al., [25]. Stirn & Hinz [58] proposed that body modifications may confer therapeutic benefits to persons with a history of self-harm. Specifically, those who reported self-cutting in childhood more frequently cited the overcoming of negative experiences as a motivation for body modification, reported a more negative body image before their modification, as well as more control over their body, feelings of self-actualisation and of being healed afterwards, compared to those who never self-harmed. A similar argument was made for the benefits of intimate piercings in persons with childhood trauma (abuse, neglect), whose body image profile was found to be comparable to data from normative samples [65].

Irrespective of trauma or self-harm, we speculate that women with navel piercings would have more body image concerns before than after their piercing, and that they would have more such concerns if they had not had their navel pierced. We therefore propose that future studies on navel piercings or other body modifications should directly measure the longitudinal aspects of women’s body image [see 66]. Future studies may also wish to systematically verify the association between body image concerns, mental health, and the number and sites of body piercings [7, 63].

### 4.3. Perception of other women’s bodies with and without piercings

Improvements in body perception extended from the self to other women’s bodies. As expected, aesthetic perception of other women’s abdomens was more favourable when the same images were shown with navel piercings than when they were shown without navel piercings, but only in women who themselves have navel piercings. This finding is in line with the assumption that participants with navel piercings would perceive them as embellishing [1, 7, 25], and therefore judge these bodies as more appealing. Other research suggests that individuals with body image disturbances give more negative ratings of body stimuli than healthy controls [67], but our study found no group differences between aesthetic ratings for bodies without navel piercings. This corroborates our earlier suggestion that there are no acute body image-related differences between women with and without navel piercings. Instead, our groups differed specifically in their aesthetic appreciation of women’s pierced abdomens.

In a task where the speeded recognition of own vs. others’ bodies was required, women with navel piercings were faster and more accurate at recognising their own body than a stranger’s body. This bodily self-advantage is similar to the processing advantage that is typically found for own vs. other faces [36] and other body parts [37–39, 68, see also 69]. Importantly, we found that the bodily self-advantage was larger for images of abdomens with navel piercings than for those without navel piercings, which again suggests that such piercings are a critical part of our sampled womens’ self-image and identity.

### 4.4. Cortical responses to (own) bodies with and without piercings

The behavioural self-advantage and aesthetic effects seen in women with navel piercings were mirrored in the electrophysiological responses. There were larger ERPs in responses to images of women’s own bodies at cortical processing stages related to early visual body-structural encoding (P1-N1 complex), identification (N250), and later emotional-motivational processing (LPP). These same three components were also modulated by group membership and the presence of piercings during the aesthetic ratings tasks. Visual ERPs to body modifications such as navel piercings, in persons with or without such modifications, have to the best of our knowledge not been reported before. In the following we will summarise and interpret our findings.

As expected, the P1-N1 complex was enhanced in both the aesthetic ratings and self-recognition tasks for bodies with piercings compared to bodies without piercings. In ratings, enhancements were found in both the pierced and control participants; however, they were strongly lateralised to the right hemisphere in pierced women (while smaller and more bilateral in controls). In the self-recognition task, a more enhanced P1-N1 complex was found for images of the self compared to images of others’ bodies. The P1-N1 self-advantage was largely restricted to the right hemisphere for images with navel piercings (while it was bilateral for images without navel piercings).

Enhancements in the P1-N1 time window indicate the greater engagement of neural resources during the observation of pierced (vs. non-pierced) bodies and of one’s own (vs. another person’s) body. ERP components P1 and N1 are related to early (ie. within 200 ms of stimulus onset) attentional processes [e.g., 44, 46] and perceptual discrimination on the basis of structural information [e.g., bodies vs. houses, 40, 41; self-face vs. other-face, 43, 70]. The presence of navel piercings likely engages attentional processes in all participants (regardless of group membership) as a result of their salience [e.g., 44], and facilitates the discriminative processing of images as (own) bodies. In pierced women, the strong lateralisation of both enhancements (pierced vs. unpierced bodies; self vs. others with piercings, but not self vs. other without piercings) to right-hemisphere posterior sites suggests that the processes of body-structural encoding and self-recognition with navel piercings are strongly interwoven and localised in right occipitotemporal cortex. Relative to its left-hemispheric homologue, this region is more strongly implicated in both body–structural encoding [e.g., 71, 72] and self-face and self-body perception [38, 39, 73, but see 37]. Early visual ERPs thus corroborate our behavioural findings that women with navel piercings have strongly integrated their piercing into their perceptual identity at the level of visual structural analysis that supports (own) body encoding and recognition.

Navel piercings did not, however, further modulate identity recognition of bodies at subsequent, intermediate stages expressed by posterior N250. The N250 is a visual ERP component at occipito-temporal sites that has been associated with the activation of stored perceptual face representations [e.g., 47, 48, 74]. Our study found that the identification of familiar (own) vs. unfamiliar (strangers’) bodies is also associated with electrophysiological enhancements in the N250 (and extending to 400ms) time window [47, 48]. However, we found no further enhancements from the presence of navel piercings, indicating that features like piercings no longer contribute to the identification of highly familiar bodies at these subsequent stages of recognition.

Both aesthetic ratings and self-recognition tasks elicited late positive potentials (LPPs) over frontal sites. The LPP typically starts from 300 ms post-stimulus and is enhanced by emotional content (both positive and negative) [e.g., 49], reflecting the sustained attentional processing of motivationally relevant stimuli [e.g., 51].

Our study found LPP enhancements for bodies with navel piercings (vs. bodies without piercings) in both aesthetic rating and self-recognition tasks. LPP enhancements for pierced bodies were more pronounced in women with navel piercings than in the control group during aesthetic ratings, similar to studies of aesthetic processing [75; for review see 50]. Roye et al., [75] showed that faces that were judged as more vs. less beautiful led to an enhanced negativity over central-parietal sites, accompanied by an enhanced positivity over frontal sites. Similarly, appetitive food stimuli enhance late positivities at frontal sites relative to neutral stimuli [51]. Our enhanced LPP for bodies with vs. without navel piercings may therefore signify positive (rather than negative) aesthetic evaluations of such bodies, especially in women who themselves have navel piercings.

LPP enhancement has also been shown for images of very underweight female bodies in adolescents with anorexia nervosa [76, 77]. In our participant sample, it may thus signify a motivated attentional narrowing toward pierced bodies in participants who have such piercings themselves. As pierced bodies enhanced LPPs in both tasks, such attentional narrowing appears to occur spontaneously, even when there are no demands to aesthetically evaluate bodies.

Frontal LPPs were also found to be enhanced for own bodies (vs. other women’s bodies) in the self-recognition task, suggesting that the sight of one’s own body also bears motivational relevance, similar to the frontal effects of self-relevant words [78]. Attentional narrowing toward the self at this stage is thought to be automatic and to play a role in self-referential processing [78]. It also occurs independently of the effects from the presence of piercings, as we found no added enhancements of the LPP for own bodies depicting navel piercings.

Put together, our electrophysiological findings show that women’s bodies with navel piercings are processed differently from those without navel piercings, both perceptually and at emotional-evaluative stages. This is likely due to the salience of piercings, which enhance the perceptual discrimination of and the sustained attention toward bodies, particularly in women who have navel piercings themselves. The ERP components related to these processes, that is, those associated with the structural analysis of bodies (P1-N1) and with positive evaluations and motivated attention toward bodies (LPP), may therefore serve as cortical markers for investigating the effects of piercings on body representations. As the same ERP components are known to be altered in women with body image disorders [e.g., 40, 76, 77], they hold promise for investigating the longitudinal effects of body piercings on body image.

### 4.5. Conclusion, limitations and constraints on generality

This study investigated for the first time how body image and body perception are affected by one type of body piercing (navel piercing) at a site that women with body image concerns commonly feel negatively about (the stomach). Our multi-method approach, which extended existing survey methods to include aesthetic ratings, perceptual responses and ERPs was able to provide an in-depth demonstration of how navel piercings change self- and other-body perception in brain and behaviour. To improve on this approach, we recommend that future studies use longitudinal designs in which they recruit participants prior to piercings and chart such perceptual, cognitive, emotional and behavioural body image changes in real time. One intriguing question such longitudinal designs may address is how the benefits from piercings or other embellishments may interact with other changes in body image and self-perception as a result of maturation and ageing, especially in those with body image concerns [e.g., 79, 80]. The prevalence of piercings at sites other than earlobes rapidly decreases after age 35 [e.g., 7, 16], suggesting that body image improvements from navel piercings may be time-limited.

Future studies investigating ERP markers would also benefit from larger sample sizes than the modest ones employed here and from the delineation of somatosensory ERPs in addition to or in combination with visual ERPs [see e.g. 81, 82] to obtain a more comprehensive picture of body-structural and -aesthetic representations at perceptual and cognitive levels in those with body piercings.

Finally, we expect our findings to generalise to other samples of (young) women who obtain navel piercings predominantly for body-aesthetic purposes within a cultural context in which body image is an important driver of self-esteem. We have no reason to believe that the results depend on other characteristics of the participants, materials or context [83].
